# Design, Preparation, and Evaluation of Enteric Coating Formulation of HPMC and Eudragit L100 on Carboxylated Agarose Hydrogel by Using Drug Tartrazine

**DOI:** 10.1155/2022/1042253

**Published:** 2022-01-28

**Authors:** Muhammad Junaid Khan, Wen-Can Huang, Muhammad Akhlaq, Sajid Raza, Alkassoumi Hassane Hamadou, Guo Yuning, Jianan Sun, Xiangzhao Mao

**Affiliations:** ^1^College of Food Science and Engineering, Ocean University of China, Qingdao 266003, China; ^2^Department of Pharmaceutics, Faculty of Pharmacy, Gomal University, Dera Ismail Khan 29050, Pakistan; ^3^Laboratory for Marine Drugs and Bioproducts of Qingdao National Laboratory for Marine Science and Technology, Qingdao 266237, China

## Abstract

Enteric-coated application on drug is used to prevent the drug from inactivation which are degraded by gastric enzyme. The present study is aimed at achieving controlled drug delivery in acidic medium of gastrointestinal tract (GIT) by enteric coating of hydroxy propyl methylcellulose (HPMC) and Eudragit L100 on carboxylated agarose hydrogel, creating a pH-dependent delivery system. Fourier-transformed infrared spectroscopy (FTIR) was for the detection of carboxylic group on agarose hydrogel, and scanning electron microscope (SEM) was used for the determination surface of prepared formulation. To check the pH sensitivity of enteric-coated formulation, different pH solution was used. It was found that the formulation was not dissolved in 1.2 but dissolve in pH 6.8 similarly; hydrogels lacking coating showed that tartrazine was more dissolved in pH 1.2, and less dissolved at pH 6.8. The release of tartrazine from the hydrogels was measured by using spectrophotometer and using a scanning electron microscope to examine the morphology and surface appearance of hydrogel capsules. This study revealed cracks on coated samples, while noncoated samples showed clear appearance with no cracks. Our findings revealed that this method could be useful for the development of an enteric coating drug delivery system.

## 1. Introduction

Controlled drug delivery is intended to provide not only continued action but also dependability, which ideally means a zero-order release rate in which the amount of drug release to the absorption site remains reasonably constant over prolong period of time [1]. This system has been used at different interval of times to deliver the drugs at different predetermined rates in order to overcome the shortcomings of ordinary drug formulations [2].

For responsive drug release in various parts of the gastrointestinal tract (GIT), the pH range of fluids may provide the environmental catalyst [3]. When specific rate control is of secondary importance, the gels can be a useful agent for pH-triggered release. The inclination of pH in the GIT increased constantly from the stomach (pH 1.2-3.5), followed by pH 5.7-6.8 along with the small intestine and colon (6.4-7.0), respectively [4]. Derivatives of cellulose and acrylic acid are commonly used as pH-sensitive polymers. The delivery system needs to be designed based on the function properties of the polymers along with their solubility property at different pH environments in order to deliver the drugs at the target sites [5].

In addition, the field of controlled drug delivery requires further development of tools for the delivery of protein and peptide drugs. In the body, the appearance of a large number of biologically active peptides is strictly controlled to maintain normal metabolic stability through a reaction called “homeostasis” [6]. If the active agent is delivered by a system that senses the signal caused by the disease, determines the signal strength, and, then takes action to release the appropriate amount of drugs, it will be very beneficial. Such a system would need to couple the drug delivery rate with physiological needs through some kind of feedback mechanism [7].

The pH range of fluids in different parts of the gastrointestinal tract (GIT) delivers environmental incentives for responsive drug release. [8] conducted studies on polymers containing weakly basic or acidic groups in the polymer backbone. The charge density of the polymer is contingent on the ion composition and pH value of the surface solution. Changing the pH of the solution will cause the polymer to swelling or deswelling. Therefore, the drug release of devices made of these polymers will show a release rate that is sensitive to pH. [9] found that the swelling characteristics of multielement gels are also affected by the composition of the buffer. A practical result suggested is that these gels may not be able to dependably facilitate pH-sensitive swelling controlled release in oral applications, because in the stomach, the level of buffer in the stomach is usually uncontrollable. [10] reported that more than two phases (collapse and swelling) can be found in gels composed of randomly distributed copolymers with negatively and positively charged groups. In these gels, the polymer segments interrelate through repulsion or attraction electrostatically and through hydrogen bonding and through repulsion or attraction electrostatic interaction.

Drug encapsulation/embedded hydrogels have different release mechanisms, such as swelling control, diffusion control, and chemical control mechanisms. The most acceptable mechanism is diffusion-controlled mechanism, and its drug release follows Fick's law of diffusion. If the molecular size of the drug molecule is much smaller than the pore size of the porous hydrogel, the porosity of the hydrogel is related to the diffusion constant of the hydrogel. When the pore size in the hydrogel is equal to the size of the drug molecule, the release of the drug molecule is delayed by the cross-linked polymer chain. As a result, the diffusion constant is reduced. If the drug release rate surpasses the swelling rate, the drug release follows the swelling control mechanism [11].

The biodegradable polymers were first used by Heller and Trescony (1979) [12]. The controlled release designs used for oral administration have been changed by pH-sensitive hydrogels. The stomach pH is less than 3, which is quite different from the rather neutral pH of intestine [13]. This pH difference is sufficient for the development of pH-sensitive polyelectrolyte hydrogels. For polycationic hydrogels, the swelling is negligible at neutral pH, and it reduces the drug release from hydrogels. This property is useful for preventing the release of foul-tasting drugs in the neutral pH environment of the mouth [14]. When tartrazine was loaded into hydrogels made of copolymers of N, N­dimethylaminoethylmethacrylate (DMAEM) and methyl methacrylate, it was not released at neutral pH. It can only be released at zero-order at pH 3-5, where DMAEM was converted into ionized form. Various types of medications include tartrazine to give a yellow, orange, or green hue to a liquid, capsule, pill, lotion, or gel, primarily for easy identification [15].

Agarose is a natural polysaccharide that consists of 1, 3-linked-*β*-d-galactose and 1, 4-linked 3, 6-anhydro *α*-l-galactose with repeating units. Agarose has been broadly used in the biomedical field, such as cell biology [16], molecular biology [17], tissue engineering [18], and drug delivery [19]. They possess low resistance to shearing, heat, and acid, and they lack active signals to animate important cell processes [20], and these characteristics have limited their functional applications. Therefore, the properties of agarose have been modified by physical, chemical, and enzymatic processes or by combinations of them.

Enteric coatings are pH-dependent, and therefore, have been used to stop gastric irritation or delay the release of drugs that are inactivated by the gastric acid in the stomach. In addition, such coatings may be applied to facilitate the delivery of a drug to its optimal absorption site in the intestine, thereby providing a delayed action, or for delivering the drug to its local site of action in the intestine [21].

Little information can be found in the literature regarding drug delivery systems and pH-sensitive drug delivery mechanisms. Considering this research gap, the present work was designed with the following objectives: (1) to test the effect of coating on carboxylated agarose hydrogel and (2) to monitor the release of tartrazine in acidic pH medium.

## 2. Materials and Methods

### 2.1. Materials

Talc (Mgso_3_), isopropanol, disodium hydrogen phosphate dodecahydrate (H_25_Na_2_O_16_P), sodium borohydride (NaBH_4_), sodium hydrogen phosphate dihydrate (Na2HPO4), sodium bromide (NaBr), and sodium chloride reagents were purchased from Sino Pharm Chemical Reagent Co. Ltd. (PR China). PEG 4000 was purchased from Solarbio, Shanghai, China, (2,2,6,6-Tetramethylpiperidin-1-yl)oxyl TEMPO was purchased from Damas-beta, Shanghai, China, sodium hypochlorite (NaOCl) was purchased from MACKLIN, Shanghai, China, and hydroxy propyl methylcellulose (HPMC) and Eudraget L 100 were purchased from Shanghai Dxiang Pharmaceutical Technology Co. Ltd.

### 2.2. Synthesis of Carboxylated Agarose

Carboxylated agarose was prepared using the protocols of [22] with slight modifications. Briefly, a 50 mL methanol solution and 2 g of native agarose (NA) were dissolved into 100 mL of beaker using a stirrer, and pH was maintained using a pH meter. The solution was heated to 80°C to dissolve agarose and then chilled at 0°C under continuous stirring to avoid gel formation of the solution. When the solution was cooled, TEMPO (0.160 mmol, 1.14 mmol), NaBr (0.9 mmol, 0.02 mmol), and NaOCl (2.5 mL, 15% vol/vol solution) were added under constant stirring. The solution was kept at a pH of 10.8 until the end of the reaction, and NaOH solution (0.5 M) was used to control the pH. At the end of the reaction, NaBH_4_ (0.1 g) was added, and the solution was acidified to pH 8 and stirred for 1 h. To precipitate carboxylated agarose (CA), NaCl (0.2 mol/L, 12 g) and ethanol (500 mL) were sequentially added. The solution was then collected using vacuum filtration and extracted by ethanol. The remaining ethanol was removed by extensive dialysis against water, and the CA was achieved as a white solid upon freeze-drying overnight. Carboxylation was confirmed using Fourier-transformed infrared spectra (FTIR) analysis.

### 2.3. Preparation of Hydrogels

CA (2% *w*/*v*) was dissolved in distilled water (15 mL) at 80°C, and 0.03 g tartrazine was added to provide a final tartrazine concentration of 0.5% *w*/*v*. Following that, 0.1 M CaCl_2_ (1 mL) was added to this solution and stirred for 20 minutes. Calcium ion was used for cross-linking on the pH release of tartrazine to be investigated. CA was designated for this study as it provides hydrogel with desired mechanical properties, where assai-low tartrazine and CaCl_2_ concentrations were chosen to decrease the potential side effects. The present study utilized Ca^2+^ ions, as they provided the best control over the release of tartrazine [22].

### 2.4. Preparation of Precoating and Overcoating Solution

Coatings on the hydrogel capsules frequently undergo insufficient bonding between the shell and the coating. Thus, previous researchers in the field of enteric coating have found that it is needed to precoat hydrogel capsules [23]. We used three different coating stages: precoating, protective coating or enteric coating, and overcoating. One accurately weighed gram of HPMC was dissolved in two-thirds of mixed solvents of ethanol and methylene chloride 20 mL (1 : 1 ratio) with stirring and was mixed for 30 minutes. Finally, the remaining solution was added into the mixture under constant stirring until the solution was clearly soluble. A similar process was followed for over coating solution.

### 2.5. Preparation of Enteric Coating Solution

Eudragit L 100 of 3 g was dissolved in 40 mL of isopropanol and acetone (1 : 1) by using magnetic stirrer. Then, PEG 4000 (14% based on the film former) and talc (6.0%) were added into the above solution with continuous stirring until the solution was fully dissolved.

### 2.6. Preparation of Eudragit L 100-Coated Capsules

CA of 0.3 g was dissolved in 15 mL distilled water at 90°C. When the CA was fully dissolved, the hydrogel solution was transferred into an ellipsoid mold to make hydrogel capsules. Uncoated capsules were placed in a Petri dish and were coated by syringe spraying. Initially, precoating solution (HPMC) was used for 30 min, and the temperature was maintained between 30 and 35°C. Next, the enteric coating solution (Eudragit L 100) was sprayed.

### 2.7. Fourier-Transformed Infrared Spectroscopy (FTIR)

Sample (1 mg) and KBr (100 mg) were crushed using a grinder and mortar and then pressed for 5 min into discs by using a 10-ton press. Fourier-transformed infrared spectroscopy (FTIR) spectra were recorded on a Vector 22 instrument (Bruker Optics), and the software provided by the manufacturer was used to analyze and import the spectrum.

### 2.8. Scanning Electron Microscope (SEM)

The morphology and surface appearance of hydrogel capsules and of coating and noncoating hydrogel capsules were examined with a scanning electron microscope (FEI Quanta FEG 650) equipped with an image analysis system. Prior to examination, the samples were gold sputter-coated to make them electrically conductive.

### 2.9. *In Vitro* Release Studies

Tartrazine 100 mL stock arrangement was set up by dissolving 10 mg of it in 100 mL of volumetric flask by utilizing phosphate buffer pH (6.8), and to make the volume upto 100 mL, more phosphate buffer was in added. The stock solution contains 0.1 mg/mL of tartrazine. In tartrazine stock solution, 50 mL solution of it was diluted in 100 mL volumetric flask by adding 50 mL of phosphate buffer (6.8 pH). The dilutions made from the stock solution contain 0.05 mg of tartyrazine, and 50 mL solution was taken in 100 mL volumetric flask, and to make the volume upto 100 mL, more phosphate buffer of (6.8 pH) were added. Each mL contains 0.025 mg of tartrazine. Similarly, dilutions of 0.0125 mg/mL, 0.00625 mg/mL, and 0.003125 mg/mL of tartrazine were prepared. Spectrophotometer (BioTek, PowerWave XS2, VT, USA) was used for analysis of tartrazine in dilute samples at 426 nm.

The release of tartrazine from the hydrogels was measured spectrophotometrically using a microplate spectrophotometer (BioTek, PowerWave XS2, VT, USA). The hydrogel precursor mixture (100 *μ*L) was added to the bottom of the 96 well plate and set at room temperature before the addition of medium (4 mL) at pH of 1.2 and 6.8. The plate was transferred to a UV spectrophotometer and set to 37°C. Time-based measurements of tartrazine release were conducted over 15, 30, 60, 120, and 180 min. The tartrazine released into the medium was determined by measuring its absorbance at 426 nm in comparison to the standard curve. The release curve was plotted as the percentage of drug released versus time.

## 3. Results and Discussion

### 3.1. pH-Responsive Hydrogel Fabrication

**Figure 1:**
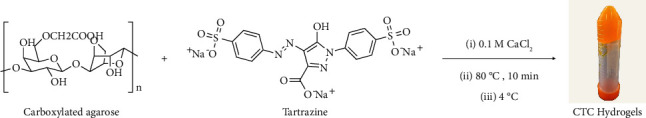
Synthesis of pH-sensitive hydrogels CTC.

pH-responsive hydrogels were prepared from carboxylated agarose, tartrazine, and CaCl_2_ and were referred to as carboxylated tartrazine calcium chloride (CTC) hydrogels (Figure 1). The CA is a derivative of agarose, which is the primary hydrogel group of the galactose units oxidized to carboxylic acid, and CA was selected for this study because of its good capability to form pH-responsive ionic interaction. Tartrazine was chosen to increase the rate of pigments. The hydrogels were prepared by dissolving CA in water at 80°C by adding tartrazine and CaCl_2_ to make hydrogel precursor solution and then cooled to 4°C to make hydrogels (Figure 1). Calcium ions were used as a cross linker to make the ionic bond between carboxylated agarose and tartrazine.

### 3.2. Enteric Coating with Release Rate

**Figure 2:**
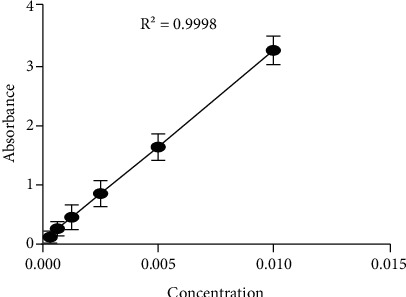
Tartrazine standard curve.

**Figure 3:**
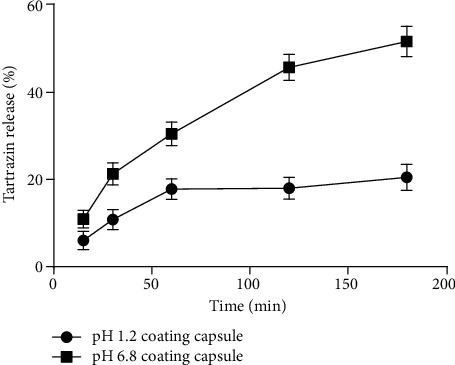
Tartrazine release rate of capsule-coated hydrogel in different pH mediums at various time intervals.

**Figure 4:**
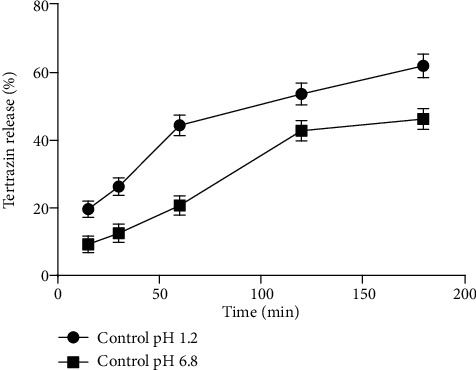
Tartrazine release rate of control hydrogels in different pH mediums at various time intervals.

Standard curve was prepared by dissolving in tartrazine in phosphate buffer with pH 7.4 and was constructed by plotting absorbance vs. concentration, and the corresponding *R*^2^ value is .9998 as shown Figure 2. The present study results showed that coated hydrogel capsules were not well dissolved in pH 1.2, but were well dissolved at pH 6.8 (Figure 3). Similarly, noncoated hydrogels showed that tartrazine is more easily dissolved at pH 1.2 and less dissolved at pH = 6.8 (Figure 4). Other studies also indicated that an enteric coating could be a polymer abstraction connected in oral medicine, enabling it to protect the drug from acidic pH environments, e.g., the stomach [24]. [25] also reported that the enteric or nonenteric hydrogels are the solutions having some other ingredients that may facilitate the application of the fabric coating. Enteric coating works with high efficiency when it is present at the surface of a highly acidic environment, such as the stomach, but breaks down quickly at a less acidic pH. For example enteric coating solution will not break up at the pH of stomach acid (1.2) but will break up in the alkaline pH (6.8) environment present in the small intestine. The dissolution of tartrazine on a coating capsule was used to evaluate the pigments on the release of tartrazine. The phosphate buffer solution is used for the dissolution experiments. pH (1.2) was used as the accelerated gastric fluid, and pH (6.8) was used as the small intestinal fluid. Initially, phosphate buffer solution was made and set in two different pHs: pH (1.2) and pH (6.8). Then, a hydrogel capsule was made, and it was coated with a Eudragit L 100 solution and HPMC.

Furthermore, HPMC was coated with hydrogel and kept for 1 hour at 35°C. Following that, HPMC coated again with Eudragit L 100 solution via syringe spraying. During the experiment for tartrazine release by using a microplate spectrophotometer (Bio Tek, Power Wave XS2, VT, USA), readings were recorded at various time intervals (15, 30, 60, 120, and 180 min).

### 3.3. Fourier-Transformed Infrared Spectroscopy (FTIR)

**Figure 5:**
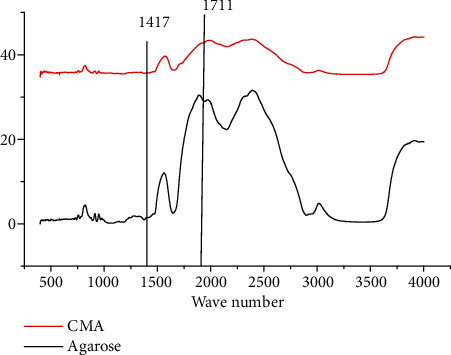
FTIR spectra of agarose and carboxylated agarose.

FTIR spectra and modified carboxylated agarose are shown in Figure 5. The agarose was compared with carboxylated agarose. The appearance of new peaks in the spectrum of the carboxylated agarose at 1417 cm^−1^ and 1711 cm^−1^ might be applied to –COO- symmetric strain and antisymmetric strain. The strains showed that there were carboxylated group in agarose chains after oxidation of TEMPO.

### 3.4. Scanning Electron Microscope (SEM)

**Figure 6:**
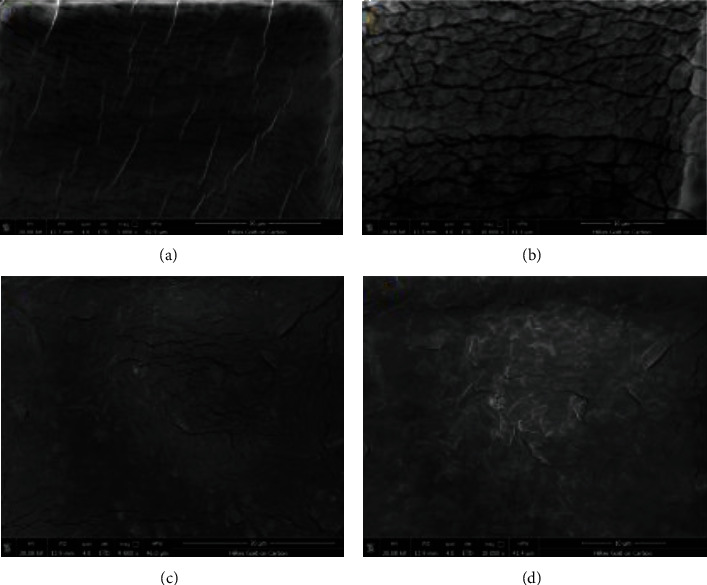
SEM image of coating (a, b) and noncoating (c, d) hydrogels.

Analysis of surface morphology was performed by scanning electron microscope with different media. The hydrogel and Eudragit L100 coating on the capsule were successfully formed. The samples that were coated developed cracks as shown in (Figures 6(a) and 6(b)). On the other hand, the samples that were not coated show no cracks and had clear appearance as shown in (Figures 6(c) and 6(d)). These results were similar to the result of [26], who found that the surface of the capsule was relatively smooth. Thus, the present results show that a good coating surface layer has smooth surface while the capsule having no surface layer represent rough surface.

## 4. Conclusion

Controlled drug delivery system is effective and most appropriate method for efficient delivery of drugs in acidic medium in GIT. In our study, tartrazine was released in stomach and intestine fluids on the acidic pH medium. This study showed the effective release of the drug in the respective targeted medium. However, on the basis of these results, it is suggested that further studies should be conducted regarding controlled drug delivery systems to strengthen the information provided in this work. Hydrogel was used as a controlled release polymer for tartrazine. FTIR were used for interaction study and SEM for coting surface. From the results of test the pHs of 1.2 and 6.8 for tartrazine release from the hydrogel, it was found that the release was appropriate at 6.8 pH.

## Data Availability

The data used to support the findings of this study are included within the article.

## Additional Points

*Highlights*. (i) Enteric-coated application on tartrazine. (ii) The drug delivery system to prevent the drug from gastric HCL. (iii) Tartrazine release was minimum at pH 1.2 and maximum at pH 6.8.
